# Comparison of Point-and-Click Performance Between the Brainfingers BCI and the Mouse

**DOI:** 10.3390/s26092777

**Published:** 2026-04-29

**Authors:** Alexandros Pino, Dimitrios Vrailas, Georgios Kouroupetroglou

**Affiliations:** Department of Informatics and Telecommunications, National and Kapodistrian University of Athens, 15784 Athens, Greece; dvrailas@di.uoa.gr (D.V.); koupe@di.uoa.gr (G.K.)

**Keywords:** brain–computer interface, human–computer interaction, pointing performance, Fitts’ law, ISO/TS 9241-411, assistive technology, pointer control

## Abstract

This study quantitatively evaluates the performance of a non-invasive hybrid brain–computer interface (BCI) compared to a conventional mouse in pointing (point-and-click) tasks. A commercial wearable BCI (Brainfingers), based on electromyography (EMG) and electrooculography (EOG) signals with low-level electroencephalography (EEG) components, was assessed against a Microsoft Optical Mouse using ISO/TS 9241-411-based one-dimensional (1D) and two-dimensional (2D) target acquisition tasks. Pointer coordinates were recorded and analyzed using Fitts’ law metrics. A total of 48 non-disabled participants completed the experiments. The results reveal significant performance differences between the two input devices. The BCI device exhibits substantially lower performance than the mouse across the reported Fitts’ law measures. Mean throughput was 0.35 bits/s for the BCI and 6.03 bits/s for the mouse in the 1D tests and 0.43 bits/s for the BCI and 5.17 bits/s for the mouse in the 2D tests. Despite the BCI’s low performance and although the present experiments involved non-disabled participants, the findings, considered alongside the prior literature on Brainfingers and non-invasive BCIs for computer access, suggest that the device may still have assistive technology value for users with severe motor impairments.

## 1. Introduction

### 1.1. Background

Brain–computer interfaces (BCIs) are a rapidly evolving area of research in the field of human–computer interaction (HCI) that aims to acquire, process, and translate neurophysiological signals into commands for operating electronic systems for communication and rehabilitation [1,2,3]. Recent research on non-invasive physiological sensing and biosignal-based interaction has reported advances in hybrid electroencephalography/electrooculography (EEG/EOG) interfaces, hybrid BCI design, EEG-based recognition and signal modeling, and low-complexity electromyography/surface electromyography (EMG/sEMG)-based wearable recognition systems [4,5,6,7]. However, these advances do not by themselves establish practical pointing performance, which remains limited by low signal amplitude, signal noise, processing latency, and user-training demands [8,9,10]. Systematic assessment of their performance in pointing (target acquisition via point-and-click) tasks is therefore crucial for evaluating their practical usefulness.

This study presents a quantitative evaluation of a commercial non-invasive hybrid BCI, compared to a conventional mouse, using a standardized framework based on Fitts’ law [11] and ISO/TS 9241-411 [12]. The novelty of the present study is not the general finding that a BCI is expected to perform worse than a conventional mouse. Rather, the contribution lies in the standardized ISO/TS 9241-411-based characterization of a specific commercial hybrid BCI system, Brainfingers, in a relatively large non-disabled sample. Brainfingers is scientifically relevant because it occupies an intermediate position between purely EEG-based BCIs and conventional muscular input devices; it uses a wearable forehead sensor configuration and transforms mixed EMG, EOG, and low-level EEG components into continuous pointer-control commands. This makes it important to quantify whether its performance is merely slow and unstable or whether it follows systematic Fitts’ law behavior under controlled pointing conditions.

The present study therefore contributes:

1. A standardized one-dimensional (1D) and two-dimensional (2D) Fitts’ law evaluation of the Brainfingers BCI under ISO/TS 9241-411-based conditions;
2. A within-participant comparison with a conventional optical mouse;
3. Regression and throughput-based characterization of Brainfingers as a pointing device;
4. Contextual comparison with previous non-invasive BCI and hybrid-interface studies relevant to computer access and assistive technology.

### 1.2. Positioning Within Relevant Literature

An early (2003) study by our Speech and Accessibility Lab examined the Brain Actuated Technologies Cyberlink system, in which “Brainfingers” referred to the control channels/software, in a small sample of non-disabled and quadriplegic users [13]. We concluded that BCI performance was significantly lower compared to a conventional mouse. Other published studies highlight ongoing challenges of BCI systems, including communication speed, signal variability, and signal stability, as well as the demanding training of users [14,15,16,17]. Standardized ISO/TS 9241-411-based evaluations of commercial hybrid BCI pointing systems remain limited.

Another early Fitts’ law-based evaluation of non-invasive BCI cursor control [18] applied a modified target-acquisition task during sensorimotor-rhythm (SMR) BCI training in non-disabled and motor-disabled participants. That study showed that Fitts’ law aptly described the relationship between movement time and index of difficulty and supported the use of information-transfer rate as a basis for comparing control modalities and participant groups on the same task [18]. Building on this line of work, ref. [19] reported mean throughput values of 0.18 bits/s, substantially below mouse performance. These findings provide an important benchmark for interpreting non-invasive BCI performance in standardized pointing tasks.

A more recent publication [20] evaluated a low-cost, single-channel EEG system designed specifically for pointer control, in which pointer movement was EEG-based and clicking was triggered by voluntary eye blinks. In a Fitts’ law-structured task, they reported an average information transfer rate (ITR) of 7 bits/min (≈0.117 bits/s), with movement-trajectory quality in certain conditions approaching that of conventional pointing devices [20]. This study demonstrates how low-cost EEG systems can be formally evaluated using Fitts’ law and highlights a significant performance gap between consumer-grade neural interfaces and standard HCI devices.

Beyond purely EEG-based systems, several hybrid BCIs that combine physiological or neural activity with eye-tracking have demonstrated enhanced performance in target-acquisition tasks. Kim et al. [21] presented a quantitative Fitts’ law comparison for a low-cost hybrid interface integrating EEG and eye movement signals. Their results showed that hybrid EEG-plus-gaze selection strategies achieved higher overall information-transfer rates than dwell-based eye tracking alone, though still below mouse-based interaction, reinforcing the notion that hybrid systems can partially mitigate the low information bandwidth of EEG control. Similarly, Hou et al. [22] evaluated a dry-electrode head-mounted sensor for visually evoked EEG interaction using Fitts’ law in a study with six participants, reporting a mean throughput of 0.82 bits/s, substantially higher than that of typical EEG pointer systems. This positions stronger hybrid or visually evoked non-invasive systems near the upper bound of what has so far been demonstrated in non-invasive BCI-driven pointing.

Within this broader landscape, the present evaluation constitutes one of the few ISO-based characterizations of Fitts’ law for a commercially available BCI. Unlike EEG systems that rely on cortical rhythms, Brainfingers exploits facial neuromuscular and ocular biosignals, enabling more stable, direct control signals under certain conditions. The standardized Fitts’ law-based evaluation presented here positions Brainfingers within contemporary BCI research and enables direct comparison with existing non-invasive BCI systems. To our knowledge, the Brainfingers BCI has not previously been evaluated under ISO/TS 9241-411-based protocols. Therefore, this work contributes a standardized, large-sample evaluation with non-disabled participants that supports reproducible benchmarking of hybrid non-invasive BCI systems.

The remainder of this paper is organized as follows. Section 2 describes the theoretical background, apparatus, participants, experimental tasks, protocol, and statistical analysis. Section 3 presents the movement time, throughput, and regression results. Section 4 discusses the findings in relation to previous work, practical usability, assistive-technology relevance, and study limitations. Section 5 concludes the paper.

## 2. Materials and Methods

In this section and throughout the rest of the paper, we use the notation and, where applicable, the definitions and terminology of ISO/TS 9241-411:2012 “Ergonomics of human-system interaction—Part 411: Evaluation methods for the design of physical input devices” [12].

**Figure 1:**
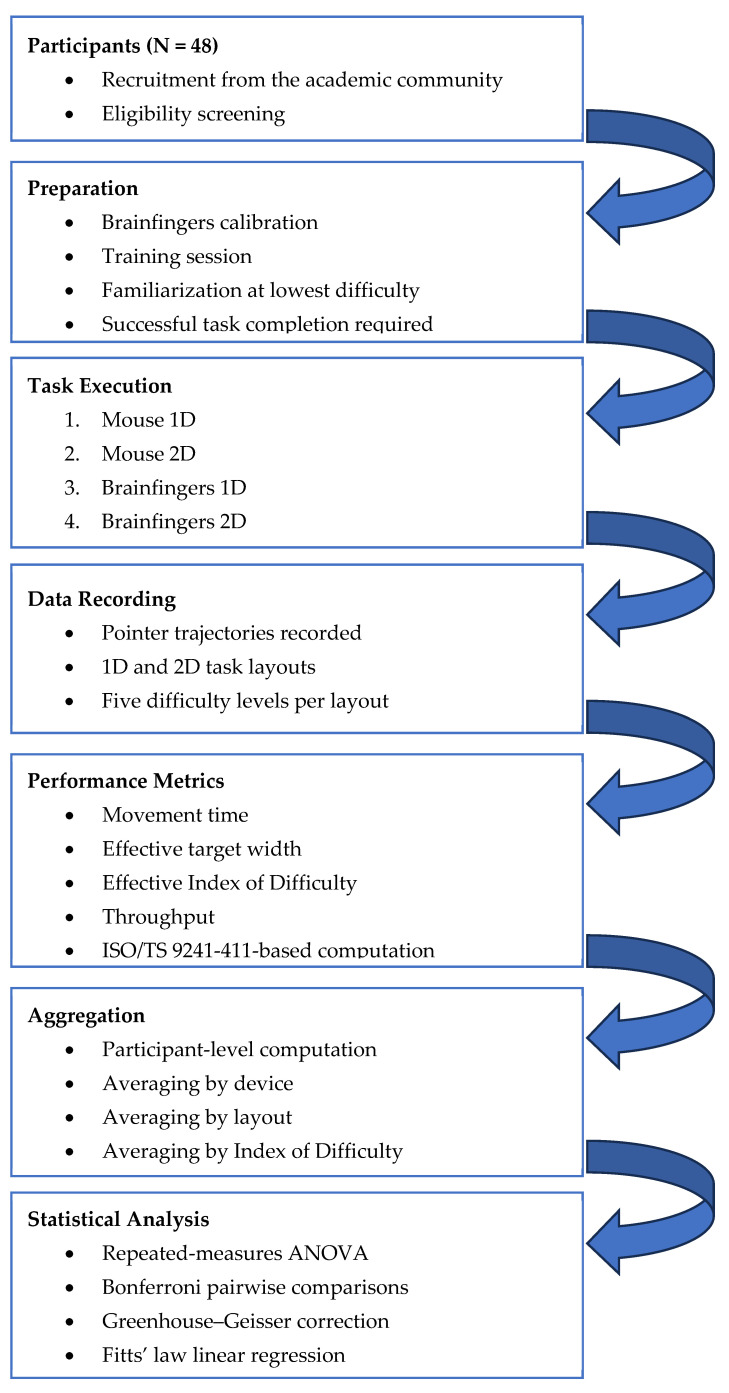
Overall workflow of the study.

Figure 1 summarizes the overall workflow of the study. Participants were recruited and screened, then completed Brainfingers calibration, training, and familiarization. In the execution phase, each participant performed 1D and 2D point-and-click tasks with the mouse and the Brainfingers BCI under the fixed experimental sequence. Pointer trajectories were recorded using the IDEA software. Movement time and effective target width were then computed, throughput was derived according to ISO/TS 9241-411, and participant-level data were analyzed using repeated-measures ANOVA and linear regression.

### 2.1. Theoretical Background

Fitts’ law [11] describes the relationship between the index of difficulty (*I*_D_) of a task, i.e., the measure of the precision required from the user in a task, and movement time (*t*_m_), i.e., the time required for task completion, calculated from the initiation of movement to target selection:

*t*_m_ = *a* + *b*·*I*_D_, (1)

where *a* (intercept) and *b* (slope) are empirical constants determined by the specific person or input device and are computed by applying linear regression to experimental data plotting *t*_m_ against *I*_D_.

The index of difficulty is measured in bits and is calculated according to the Shannon formulation [23] by:

*I*_D_ = log_2_ (*d*/*w* + 1), (2)

where *d* is the distance of movement to the target and *w* is the target width of the displayed target along the approach axis for pointing tasks. In the 1D task of the present study, this operational definition was applied using circular targets, with *w* taken as the target diameter along the horizontal movement axis.

ISO/TS 9241-411 defines how to assess pointing actions on a graphical user interface (GUI) based on Fitts’ law. It standardizes the target selection tests with one-dimensional (1D) and two-dimensional (2D) target layouts [12]. The tests provide a measure of throughput, namely the rate of information transfer when a user operates an input device to control a pointer on a display; throughput is expressed in bits per second (bits/s). *t*_m_ and throughput derived from Fitts’ law estimate the speed, accuracy, and overall efficiency of each input device. The following calculations are for input throughput for pointing tasks:

Throughput = Effective index of difficulty/Movement time = *I*_De_/*t*_m_, (3)

where the effective index of difficulty (*I*_De_) is the measure, in bits, of the user precision achieved in accomplishing a task expressed as:

*I*_De_ = log_2_ (*d*/*w*_e_ + 1), (4)

where the effective target width (*w*_e_) is the width of the distribution of selection coordinates made by a subject during a pointing test. It is calculated as:

*w*_e_ = 4.133·*s*_x_ (5)

where *s*_x_ is the standard deviation of the selection coordinates in the direction of movement (e.g., x-axis in a horizontal pointing test).

### 2.2. Apparatus

The experimental setup was developed at the Voice and Accessibility Laboratory of the Department of Informatics and Telecommunications of the National and Kapodistrian University of Athens. The evaluated device was the Brainfingers system (Brain Actuated Technologies, Inc., Yellow Springs, OH, USA), consisting of an adjustable forehead headband with six dry-contact frontal electrodes, an external amplifier/processing unit, and accompanying software. According to the developer’s technical documentation, the forehead signal contains overlapping EOG, EMG, and EEG components. The six-electrode headband produces a single composite signal, which the Brainfingers software (version 11.10) processes into multiple control channels, including Glance, Theta, Alpha, Beta, and Muscle signals [24]. In this sense, Brainfingers should be interpreted in the present study as a commercial, non-invasive hybrid brain–body interface rather than a purely EEG-based BCI. A Microsoft Basic Optical Mouse (Microsoft Corporation, Redmond, WA, USA) was used as the reference input device for performance comparison.

Because Brainfingers is a closed commercial system that internally transforms the composite forehead signal into control channels, this study did not include offline preprocessing, artifact removal, feature extraction, or machine-learning classification of raw EEG, EMG, or EOG signals. The analyzed data consisted of the resulting pointer trajectories recorded during task execution.

The tests were performed on a desktop computer running Windows 10, equipped with a 24-inch LCD monitor (1920 × 1080, 60 Hz). The target layouts for the 1D and 2D tasks were implemented in accordance with ISO/TS 9241-411, with the exact task geometry and operational definitions described in the next sections. Pointer trajectories were automatically acquired as a series of pointer coordinates in pixels and analyzed. For the graphical environment of the experiments, data acquisition, and pointer-trajectory analysis, our lab’s (Voice and Accessibility Laboratory, Athens, Greece) proprietary Input Device Evaluation Application (IDEA) software was used. Published studies over the last two decades demonstrate the validity of the IDEA software in multiple experiments [13,25,26,27,28,29,30,31].

### 2.3. Participants

A total of 48 adults participated in the study, recruited from the academic community (undergraduate and postgraduate students at the Department of Informatics and Telecommunications of the National and Kapodistrian University of Athens). The sample comprised 28 men (58.3%) and 20 women (41.7%), with ages ranging from 21 to 48 years (mean age = 30.29 years, standard deviation (SD) = 7.03; median = 30 years). Participation criteria required that participants:

- Possessed a high level of computer familiarity;
- Had no prior experience in using a BCI;
- Had no diagnosed disabilities in the upper limbs;
- Had no visual disabilities;
- Completed the BCI Brainfingers familiarization session successfully.

No formal a priori power analysis was conducted. The sample size was determined by participant availability within the academic setting and by the practical duration of the Brainfingers calibration, training, and testing protocol. Nevertheless, the final sample of 48 participants compares favorably with several previous experimental studies of non-invasive BCI-based pointing and hybrid target-selection systems, including studies with five, six, or small mixed participant samples [13,19,22]. Therefore, it was considered adequate for a within-participant exploratory benchmarking study of this device.

None of the participants had any disability (e.g., hearing or dexterity impairment) or a learning difficulty. All participants were informed about the study’s purpose and received explanatory instructions for all stages and tests. They all confirmed that they fully understood the experimental procedure of the current study, and written informed consent was obtained from all participants in printed form. The research followed the tenets of the Helsinki Declaration and was approved by the Ethics Committee of the National and Kapodistrian University of Athens. Participation was voluntary. No raw biosignals were stored at any stage of the experiments. Only pointer trajectories were recorded and used in the current study.

### 2.4. Experimental Tasks

**Figure 2:**
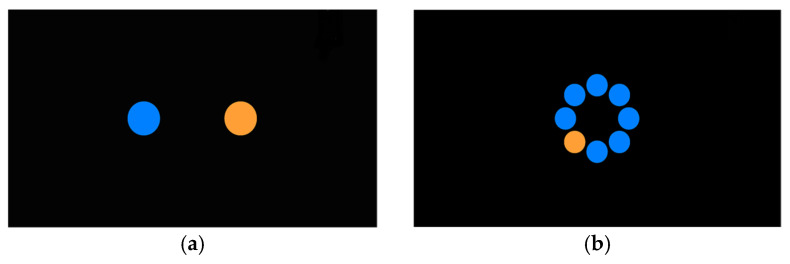
Target layouts at lowest indexes of difficulty: (**a**) 1D experiment screen; (**b**) 2D experiment screen. Blue circles indicate the start or non-destination targets, and orange circles indicate the destination target.

Each participant completed the required target acquisition tasks with the mouse and the BCI in both 1D and 2D environments. Two typical target layouts are shown in Figure 2.

#### 2.4.1. One-Dimensional (1D) Pointing Task

During the 1D tasks, two circular targets were displayed along the horizontal axis. Although ISO/TS 9241-411 defines a target width for pointing tasks along the approach axis, it does not prescribe a unique orthogonal target geometry for 1D graphical targets. In the present study, circular targets were used in the 1D task because pointer motion on the display was not mechanically constrained to a single axis for either device. With the Brainfingers BCI in particular, it was difficult to maintain strictly horizontal trajectories throughout target acquisition. Under these conditions, short rectangular targets would introduce an arbitrary orthogonal tolerance through target height and could materially affect acquisition difficulty. Circular targets reduce this geometric confounding while preserving a well-defined 1D width parameter. Accordingly, in the 1D task, *w* was defined as the target diameter, i.e., the extent of the target along the horizontal movement axis. This treatment is consistent with prior analyses showing that circular targets preserve the 1D interpretation better than rectangular targets when the approach angle varies and with later work that explicitly employed circular targets in reciprocal 1D pointing tasks [32,33,34].

**Figure 3:**
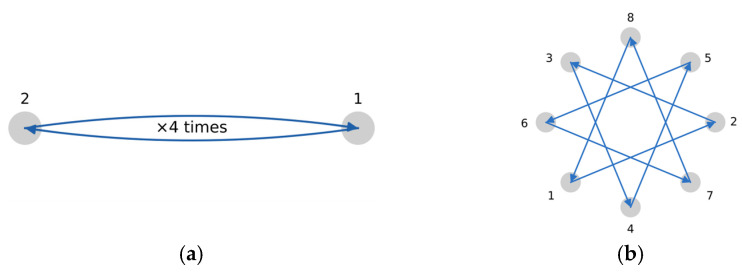
Selection sequence pattern in the (**a**) 1D experiment and (**b**) 2D experiment. Targets are numbered in the order they should be selected.

Targets were colored blue (designating the start target) and orange (designating the destination target). Participants were instructed to move the pointer from the center of the left (blue) target, where it initially appeared, to the right target and select it as accurately as possible. After each successful click, the targets toggled colors, and the participants had to start from the center of the right target, where the pointer was automatically located, to the left target and click on it. Each attempt, either from left to right or from right to left, is defined as a “trial”. Figure 3a is a schematic representation of the 1D reciprocal target selection sequence. One left-to-right and one right-to-left movement are shown; the pair was repeated four times, yielding eight trials in total. After completing eight trials successfully, the distance *d* between the targets increased, and the target width *w*, namely the diameter, decreased, yielding a new *I*_D_. Each set of eight trials with an unchanged *I*_D_ is defined as a “session”. We use five indexes of difficulty, namely five combinations of *d* and *w*, for a total of 40 trials across five sessions to complete the 1D test.

#### 2.4.2. Two-Dimensional (2D) Pointing Task

During the 2D tests, eight targets were displayed in a circular layout. The use of circular targets in the 2D task is consistent with established multidirectional Fitts’ law paradigms, in which movement amplitude is defined by the center-to-center distance between successive targets, and target width *w* is measured as the target diameter [32,33]. However, recent methodological analysis has shown that, in the canonical ISO-style multidirectional sequence, equal movement distance across a sequence is guaranteed only when an odd number of targets is used; with an even number of targets, a nearly diametrically opposed sequence produces two alternating movement distances across trials [35]. Because the present study used eight targets, the selection sequence was implemented as a fixed, clockwise, constant-step pattern (Figure 3b), beginning at the top target, so that all successive movements had the same center-to-center distance. This sequencing choice was a deliberate ISO-based adaptation intended to preserve constant movement distance across trials while retaining an eight-target layout [35]. An odd number of targets would also have avoided alternating movement distances in the canonical sequence; however, the eight-target layout was retained because it had already been implemented in the IDEA experimental environment and used in the original data-collection protocol. The sequencing adaptation was therefore applied to preserve constant center-to-center movement distance within this fixed layout.

All targets were colored blue, except the destination target in each trial, which was colored orange. After the successful completion of each session, namely the eight trials, the target diameter is decreased and the center-to-center distance between successive targets is increased, yielding a higher *I*_D_ for the next session. Five indexes of difficulty are used, resulting in 5 sessions and a total of 40 trials, also for the 2D experiment.

### 2.5. Experimental Protocol

The experiment consisted of two main phases, each taking place on a different day with a one-week interval: Training and Familiarization, and Execution.

#### 2.5.1. Training and Familiarization

At this stage, the Brainfingers operation principles and the structure of the experimental tasks were explained to all participants. Emphasis was given on how to achieve click commands and control the pointer. The process was supported by the features and procedures included in the Brainfingers software. For each participant, a calibration procedure lasting approximately 10 min was conducted, in which participants performed a series of guided actions to confirm control and ensure reliable biosignals detection.

In the training session that followed, lasting approximately 1 h, the participants completed the following tasks:

- Basic pointer control (jaw tension for clicks, forehead tension/relaxation for up/down pointer movements, and eye glimpses/relaxation for right/left pointer movement);
- Repeated attempts to stabilize control thresholds, based on real-time feedback;
- Target acquisition actions to ensure that the signal strength and threshold values consistently respond to activation commands.

The calibration and training sessions were necessary to ensure stable biosignal acquisition and to help the user learn how to limit undesirable EMG/EOG activity.

After a half-hour break, a familiarization session followed, during which each participant successfully performed the experimental tasks at the lowest *I*_D_ level in the 1D and 2D experiments.

#### 2.5.2. Execution

All tasks were performed with participants seated at a desk in an ergonomically defined position, maintaining a fixed viewing distance of approximately 50 cm from the monitor. Experimental procedures took place under strictly defined laboratory conditions at the Voice and Accessibility Laboratory. The execution phase lasted as long as each participant needed to complete both 1D and 2D experiments, at five indexes of difficulty for each test, with both Brainfingers BCI and the mouse (a total of 160 trials).

Device order and task order were fixed for all participants:

1. 1D test with the mouse.
2. 2D test with the mouse.
3. 1D test with the Brainfingers BCI.
4. 2D test with the Brainfingers BCI.

### 2.6. Analysis

Statistical analysis was performed at the participant level. For each participant, movement time (*t*_m_) was first computed for each successful trial and then averaged for each combination of device, task layout, and index of difficulty (*I*_D_). Throughput was computed using the effective index of difficulty (*I*_De_) divided by the corresponding movement time, following the ISO/TS 9241-411 formulation. For each participant and condition, throughput values were then averaged across the trials belonging to the same device, task layout, and *I*_D_ level. Thus, the inferential analyses were based on participant-level mean values rather than on pooled trial-level observations.

Because the same participants completed all device and *I*_D_ conditions, a repeated-measures design was used. The two within-subject factors were device (Brainfingers BCI and mouse) and *I*_D_ (five levels). Because the 1D and 2D tasks represent different target-acquisition layouts, inferential analyses were conducted separately for each layout. For movement time and throughput, two-way repeated-measures analyses of variance (ANOVAs) were performed separately for the 1D and 2D tasks. This approach was selected because it allows simultaneous assessment of the main effect of device, the main effect of task difficulty, and the device × *I*_D_ interaction within the same participants.

Sphericity for effects involving *I*_D_ was assessed with Mauchly’s test; when violated, Greenhouse–Geisser correction was applied. For significant interactions, follow-up pairwise comparisons between devices at each *I*_D_ level were performed with Bonferroni adjustment. Effect sizes are reported as partial eta squared (ηp^2^). The level of statistical significance was set at *p* < 0.05. In addition, linear regression of mean movement time against the index of difficulty was performed for each device and task layout to examine conformity with Fitts’ law.

This inferential-analysis approach is consistent with prior Fitts’ law-based evaluations of alternative pointing and assistive access devices, which have also used ANOVA-based comparisons of movement time and/or throughput across devices and task conditions [36,37].

## 3. Results

This section presents the quantitative results of the execution phase, based on participants’ overall performance in the 1D and 2D target-acquisition tasks across both devices and all tested indexes of difficulty. All descriptive results represent means and standard deviations across participants, based on each participant’s values for each device, task layout, and index of difficulty. The results’ interpretation is provided in the Discussion section.

### 3.1. Movement Time

**Figure 4:**
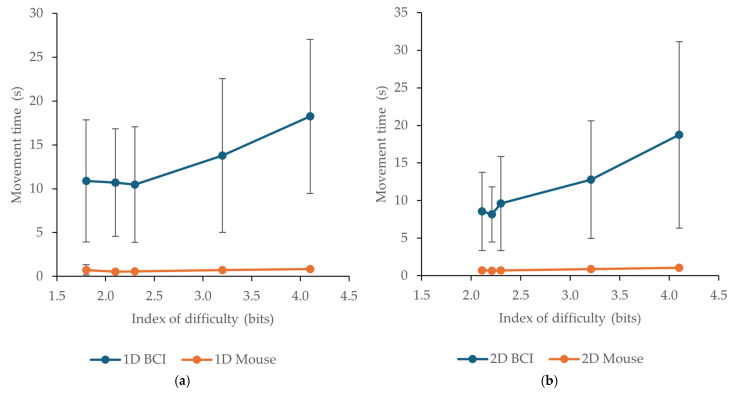
Mean movement time *t*_m_ across tested indexes of difficulty *I*_D_ for the BCI and the mouse: (**a**) 1D tests; (**b**) 2D tests. Error bars (where visible) indicate standard deviations (SD).

Mean movement time values for the Brainfingers BCI and the mouse across the tested indexes of difficulty are presented in Table 1 and Figure 4. In both task layouts, the mouse yielded substantially lower movement times than the BCI at all tested difficulty levels. In the 1D task, mouse *t*_m_ ranged from 0.55 s to 0.86 s, whereas the corresponding BCI values ranged from 10.48 s to 18.25 s. In the 2D task, mouse *t*_m_ ranged from 0.66 s to 1.06 s, while BCI *t*_m_ ranged from 8.16 s to 18.73 s. Thus, across all tested IDs, target acquisition with the Brainfingers BCI was markedly slower than with the conventional mouse.

For both devices, movement time generally increased with task difficulty. This trend was particularly clear for the BCI, especially at the highest *I*_D_ values, where movement times rose considerably in both 1D and 2D tasks. The same overall pattern was observed for the mouse, although the absolute increase in *t*_m_ was much smaller. Variability was also notably higher for the BCI, as reflected by the larger standard deviations reported in Table 1 and the more pronounced error bars in Figure 4. Overall, these results indicate a substantial performance gap between the two devices in terms of speed of target acquisition, with the gap becoming more evident as task difficulty increased.

Inferential analysis confirmed these patterns. For movement time in the 1D task, repeated-measures ANOVA showed significant main effects of device, F(1, 47) = 176.77, *p* < 0.001, ηp^2^ = 0.79, and *I*_D_, F(4, 188) = 28.23, *p* < 0.001, ηp^2^ = 0.38, as well as a significant device × *I*_D_ interaction, F(4, 188) = 24.31, *p* < 0.001, ηp^2^ = 0.34. For the 2D task, there was again a significant main effect of device, F(1, 47) = 173.78, *p* < 0.001, ηp^2^ = 0.79. After Greenhouse–Geisser correction, the main effect of *I*_D_, F(2.07, 97.41) = 30.32, *p* < 0.001, ηp^2^ = 0.39, and the device × *I*_D_ interaction, F(2.06, 96.75) = 26.00, *p* < 0.001, ηp^2^ = 0.36, also remained significant. Bonferroni-adjusted pairwise comparisons showed that the mouse yielded significantly lower movement times than the Brainfingers BCI at every tested *I*_D_ in both task layouts (all *p* < 0.001).

### 3.2. Throughput

**Figure 5:**
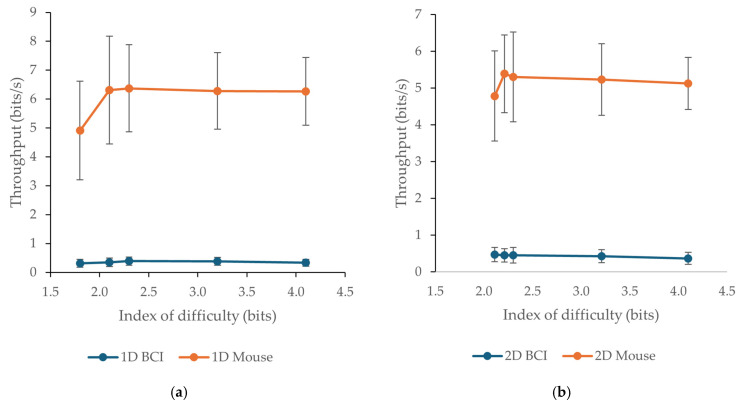
Mean throughput chart across tested indexes of difficulty *I*_D_ for the BCI and the mouse: (**a**) 1D tests; (**b**) 2D tests. Error bars indicate standard deviations.

Mean throughput values for the Brainfingers BCI and the mouse across the tested indexes of difficulty are presented in Table 2 and Figure 5. The mouse consistently achieved much higher throughput than the BCI in both task layouts and at all tested levels of difficulty. In the 1D task, mouse throughput ranged from 4.91 to 6.37 bits/s, whereas BCI throughput ranged from 0.32 to 0.39 bits/s. In the 2D task, mouse throughput ranged from 4.78 to 5.39 bits/s, while BCI throughput ranged from 0.37 to 0.47 bits/s. These values indicate a markedly lower rate of information transfer for the BCI compared with the mouse.

The mouse maintained high throughput across the tested indexes of difficulty, with only limited variation, particularly in the 1D task. In contrast, BCI throughput remained low, showing only small changes across difficulty levels. In the 2D task, BCI throughput decreased slightly as the index of difficulty increased, while mouse throughput also showed a modest downward trend at higher indexes of difficulty. Overall, the throughput results are consistent with the movement time findings and confirm the substantially lower efficiency of the Brainfingers BCI as a pointing device under the present experimental conditions.

Inferential analysis also supported the throughput findings. For the 1D task, repeated-measures ANOVA showed a significant main effect of device, F(1, 47) = 1471.09, *p* < 0.001, ηp^2^ = 0.97. After Greenhouse–Geisser correction, both the main effect of *I*_D_, F(3.24, 152.11) = 13.14, *p* < 0.001, ηp^2^ = 0.22, and the device × *I*_D_ interaction, F(3.32, 156.00) = 11.27, *p* < 0.001, ηp^2^ = 0.19, also remained significant. For the 2D task, significant main effects were again found for device, F(1, 47) = 1967.62, *p* < 0.001, ηp^2^ = 0.98, and *I*_D_, F(4, 188) = 3.92, *p* = 0.004, ηp^2^ = 0.08, as well as for the device × *I*_D_ interaction, F(4, 188) = 4.34, *p* = 0.002, ηp^2^ = 0.08. Bonferroni-adjusted pairwise comparisons showed that the mouse yielded significantly higher throughput than the Brainfingers BCI at every tested *I*_D_ in both task layouts (all *p* < 0.001).

### 3.3. Linear Regression of Movement Time on Index of Difficulty

**Figure 6:**
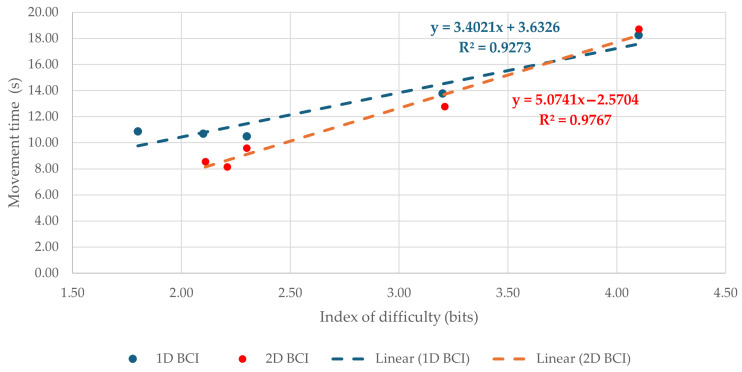
Linear regression of movement time, *t*_m_, on the index of difficulty, *I*_D_, for the Brainfingers BCI in the 1D and 2D tasks.

**Figure 7:**
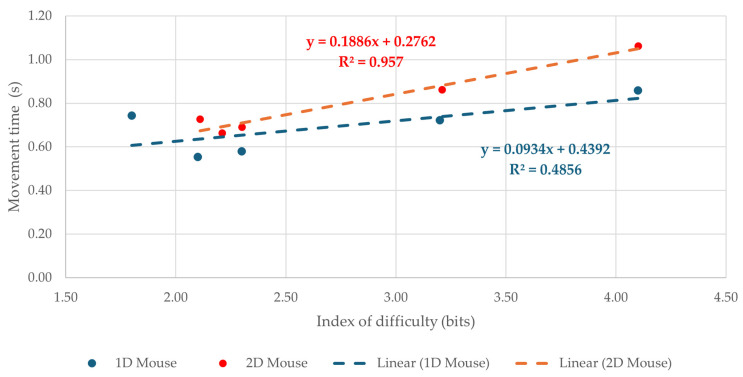
Linear regression of movement time, *t*_m_, on the index of difficulty, *I*_D_, for the mouse in the 1D and 2D tasks.

Linear regression was applied to the mean movement time values across all tested *I*_D_s to examine the relationship between *t*_m_ and task difficulty. The regression plots are shown in Figure 6 for Brainfingers and Figure 7 for the mouse. For the Brainfingers BCI, the fitted models were *t*_m_ = 3.63 + 3.40·*I*_D_ for the 1D task and *t*_m_ = −2.57 + 5.07·*I*_D_ for the 2D task, with R^2^ = 0.93 and R^2^ = 0.98, respectively. For the mouse, the fitted models were *t*_m_ = 0.44 + 0.09·*I_D_* for the 1D task and *t*_m_ = 0.28 + 0.19·*I*_D_ for the 2D task, with R^2^ = 0.49 and R^2^ = 0.96, respectively.

In both devices, movement time increased with increasing *I*_D_, but the fitted parameters differed substantially between devices and task layouts. The Brainfingers BCI showed steeper slopes than the mouse in both 1D and 2D tasks, indicating a stronger increase in movement time with increasing task difficulty. The 2D BCI condition produced the steepest slope of all four cases (*b* = 5.07), consistent with the particularly demanding nature of multidirectional target acquisition using the BCI. The regression fit was strong for the BCI across both task layouts and for the mouse in the 2D task. In contrast, the mouse 1D condition showed a lower R^2^, likely due to the very small absolute range of *t*_m_ values across *I*_D_s. Overall, the regression results suggest an approximately linear relationship between movement time and index of difficulty, while also highlighting the substantially poorer efficiency of the BCI compared with the mouse. However, because each regression was fitted on five *I*_D_ levels, the slope and R^2^ values should be interpreted as descriptive indicators of Fitts’ law consistency rather than as robust model-validation evidence.

## 4. Discussion

The present study showed a clear and consistent performance gap between the Brainfingers BCI and the conventional mouse across one- and two-dimensional pointing tasks. Across all tested indexes of difficulty, the mouse produced substantially lower movement times and substantially higher throughput values than the BCI. The inferential analysis confirmed that these differences were not limited to isolated conditions but also reflected strong overall device effects and significant device × *I*_D_ interactions. Thus, the lower performance of the Brainfingers BCI reflected not only reduced absolute speed but also greater sensitivity to increasing task difficulty. In practical terms, the present results indicate that Brainfingers, in its current form and under the present experimental conditions, cannot be considered comparable to a conventional mouse for general-purpose point-and-click interaction.

The practical significance of this difference is larger than the statistical comparison alone indicates. Mouse throughput values around 5–6 bits/s reflect efficient everyday pointing performance, whereas Brainfingers throughput remained below 0.5 bits/s in both task layouts. In practical terms, Brainfingers required many seconds for target selections that the mouse completed in less than one second. Therefore, under the present conditions, Brainfingers should not be interpreted as a practical substitute for a mouse in general computer use. Its relevance is instead restricted to assistive-access scenarios in which conventional hand-operated pointing devices are unavailable or unusable. No universal throughput threshold defines acceptability for assistive computer access, because usability depends strongly on the user’s residual motor abilities, available alternatives, task demands, and training duration.

At the same time, the regression analysis showed that movement time increased systematically with the index of difficulty for the Brainfingers BCI in both task layouts, with strong coefficients of determination. This is an important finding because it indicates that the BCI did not behave as an erratic or purely unstable controller but rather as an input system whose performance can be meaningfully characterized within the Fitts’ law framework [11,12]. This observation is in line with earlier studies showing that non-invasive BCI cursor-control performance can be meaningfully characterized within a Fitts’ law framework, including modified target-acquisition tasks in non-disabled and motor-disabled users [18] and later 2D EEG-based pointing evaluations [19]. Therefore, despite its markedly lower efficiency compared with the mouse, Brainfingers showed evidence of structured, quantifiable behavior under standardized pointing conditions. However, as noted above, this interpretation should remain cautious because the regression analysis was based on five *I*_D_ levels per condition.

The relationship between the 1D and 2D findings also deserves careful interpretation. For the mouse, performance was broadly consistent with the established multidirectional pointing literature, with slightly higher and more stable throughput in the 1D task than in the 2D task [32,33]. For the Brainfingers BCI, however, the pattern was less straightforward. Although the mean 2D throughput values were slightly higher than the corresponding 1D values at some lower IDs, the 2D regression slope was steeper, and movement time increased markedly at higher difficulty levels. Therefore, the 2D task should not be interpreted as generally easier for the BCI. Rather, the observed pattern likely reflects the combined influence of target geometry, effective width estimation, control strategy, and variability in biosignal-driven pointer trajectories. The particularly steep 2D slope suggests that multidirectional pointing remained especially demanding for the BCI as task difficulty increased.

A useful historical point of comparison is our earlier study on the Brain Actuated Technologies Cyberlink system, which also adopted an ISO-based evaluation framework and compared non-disabled and motion-impaired users performing point-and-click tasks with the BCI and a mouse [13]. In that earlier work, the BCI was again found to be clearly inferior to the mouse in terms of usability. Nevertheless, it was considered a possible alternative when conventional hand-actuated input was not feasible. However, unlike the present study, the earlier data did not support a clear Fitts’ law fit for BCI performance. This difference should be interpreted cautiously, because the two studies are not directly equivalent in design; the earlier work involved a much smaller mixed sample of non-disabled and motion-impaired users, older ISO 9241-9-based task implementations, and different analytical emphases, including detailed cursor measures and learning-related observations. Nevertheless, taken together, the two studies suggest a consistent overall conclusion that these Brain Actuated Technologies hands-free systems remained markedly inferior to a mouse for general-purpose pointing. At the same time, the present results extend the earlier work by showing that, under a more standardized contemporary protocol and with a larger non-disabled sample, the current Brainfingers system can still be characterized in a structured way within the Fitts’ law framework. According to personal communication with their principal developer, the later Brainfingers product was considered the next model in this product line.

In relation to the previous literature, the throughput values observed for Brainfingers place the device within the broader low-bandwidth range of non-invasive BCI-based pointing systems. The present values are higher than those reported in some earlier non-invasive BCI pointing studies, such as the 2D EEG-based BCI in [19] and the single-channel NeuroSky-based pointer-control system in [20]. However, the latter comparison should be interpreted cautiously, as [20] reports an estimated information-transfer rate rather than directly comparable ISO-style pointing throughput. At the same time, the present values remain below the performance reported for stronger hybrid or visually evoked approaches [21,22]. This comparison is plausible given Brainfingers’ hybrid nature. Unlike purely EEG-based systems, Brainfingers relies heavily on voluntary facial, neuromuscular, and ocular biosignals, which can provide more robust control than cortical rhythms alone but still do not approach the continuous, high-precision motor control afforded by a conventional mouse.

Table 3 summarizes reported information-transfer values for the present study and selected related studies already cited in this paper. The table is intended solely as a contextual benchmark, as participant groups, task implementations, device types, and metric definitions are not fully equivalent across studies. The studies were selected because they are directly relevant to non-invasive BCI or hybrid target-acquisition/computer-access tasks and because they report information-transfer or throughput-type values that can at least partially contextualize the present results; the table is not intended as a systematic review.

As shown in Table 3, the present Brainfingers values are higher than those reported in some earlier non-invasive BCI pointing studies, such as the 2003 Cyberlink system in non-disabled users [13], the 2D EEG-based BCI in [19], and the single-channel NeuroSky-based approach in [20]. However, the latter comparison should be interpreted cautiously because [20] reports estimated ITR rather than directly comparable ISO-style throughput. At the same time, the present values remain below those reported for stronger hybrid or visually evoked approaches [21,22]. Whenever a directly reported conventional comparator is available, mouse performance remains substantially higher than BCI or hybrid control [13,19,20].

From an assistive-technology perspective, the present findings should be interpreted with caution. The participants in this study were non-disabled and, therefore, the results do not directly demonstrate clinical effectiveness or practical benefit for users with severe motor impairments. However, this does not make the findings irrelevant to assistive technology. For individuals who cannot use a conventional mouse at all, the relevant question is not whether a BCI matches mouse performance but whether it can provide a functional channel for computer access. The prior literature has established the broader relevance of non-invasive BCIs for communication, augmentative and alternative communication (AAC), environmental control, and computer access in populations with severe disabilities [38,39,40,41]. Our earlier Brain Actuated Technologies study [13] also supported the view that such systems may retain assistive value even when they remain clearly inferior to a mouse. At the same time, the clinical and review literature suggests both promise and substantial variability. For example, Cincotti et al. [38] described an integrated assistive prototype for users with severe motor disabilities, combining several access technologies, and four participants learned to operate the system through a non-invasive EEG-based BCI. More recently, a systematic review of AAC-BCI research for individuals with disabilities concluded that such systems show promise for communication access but remain ineffective for some users and exhibit substantial variability in performance and reporting practices [41]. Accordingly, the present study should be viewed as a device characterization and benchmarking study that informs the assistive potential of Brainfingers, rather than as a direct demonstration of clinical usability.

### 4.1. Limitations

Several limitations of the present study should be acknowledged. First, device order and task order were fixed for all participants, with the mouse always preceding the BCI and the 1D task always preceding the 2D task. Therefore, device-related differences cannot be interpreted independently of possible order, learning, transfer, or fatigue effects. We did not perform an early-versus-late trial analysis because the trials were not repeated under identical conditions; as the experiment progressed, the index of difficulty increased, so later trials were also harder trials. This limitation is particularly important because the mouse was a highly familiar device for all participants, whereas Brainfingers required new biosignal-control strategies. Consequently, the observed BCI performance may reflect short-term familiarization performance rather than stable performance after extended practice. Future studies should use counterbalanced device/task orders and longer longitudinal training protocols to estimate learning effects and asymptotic BCI performance more reliably. Second, the tested indexes of difficulty were not evenly distributed across the examined range, with denser sampling at lower *I*_D_ values than at intermediate and higher values. Therefore, local fluctuations among the lower *I*_D_ conditions should be interpreted cautiously, because the visual shape of the plotted curves may overemphasize small irregularities in that region, even though the overall inferential and regression results still support the broader performance trends. Third, Brainfingers is a hybrid interface that relies heavily on EMG and EOG components, so the findings should not be generalized to purely EEG-based BCIs. Because Brainfingers is a closed commercial integrated system, the separate contribution of individual EEG, EMG, and EOG components could not be isolated experimentally in the present study. Therefore, modality-specific ablation analysis was outside the scope of this work. Finally, the relatively large standard deviations across several BCI conditions indicate substantial inter-participant variability, consistent with the broader BCI literature and with systematic review evidence showing that AAC-BCI performance remains highly variable across users with disabilities [39,41,42].

### 4.2. Future Work

Future work should include users with motor impairments, counterbalanced experimental designs, more evenly distributed *I*_D_ levels, and longer-term training protocols to determine the extent of achievable performance improvement in realistic assistive scenarios.

In addition, future work should include explicit error-related and trajectory-based performance measures beyond movement time and throughput. Although the present study incorporated accuracy indirectly through the effective target width and effective index of difficulty used in the throughput calculation, dedicated measures such as missed clicks, target re-entries, task-axis crossings, movement direction changes, orthogonal direction changes, movement offset, movement error, and movement variability may provide a more fine-grained characterization of control quality and error patterns [43,44]. Such measures could help clarify how and why Brainfingers performance differs from mouse performance beyond the differences already captured by movement time and throughput.

## 5. Conclusions

This study provided a standardized quantitative comparison between the Brainfingers non-invasive hybrid BCI and a conventional mouse in ISO/TS 9241-411-based 1D and 2D point-and-click tasks. Mean throughput was 0.35 bits/s and 0.43 bits/s for Brainfingers in the 1D and 2D tasks, respectively, compared with 6.03 bits/s and 5.17 bits/s for the mouse. Across all tested indexes of difficulty, the mouse consistently outperformed the BCI, yielding substantially lower movement times and substantially higher throughput in both task layouts. Inferential analysis confirmed significant effects of device and task difficulty, as well as significant device × *I*_D_ interactions, indicating that the performance gap was robust across conditions. At the same time, the Brainfingers BCI showed an approximately linear increase in movement time with increasing index of difficulty, suggesting that the Fitts’ law framework can be used descriptively to characterize its performance. Therefore, although Brainfingers cannot be considered comparable to a conventional mouse for general-purpose pointing interaction under the present conditions, it still demonstrated structured and quantifiable behavior as an input device. Given that conventional pointing devices may be unusable for some end users and considering the broader literature on non-invasive BCIs and assistive technologies for computer access [38,39,41,45,46], the present findings support continued investigation of Brainfingers as a potential assistive technology tool. Future work should evaluate Brainfingers in target populations with motor impairments under more rigorous and longer-term experimental conditions.

## Figures and Tables

**Table 1:** Mean movement time *t*_m_ (s) and standard deviation (in parentheses) across tested indexes of difficulty *I*_D_ (bits) for the BCI and the mouse at 1D and 2D tests.

1D	BCI	Mouse	2D	BCI	Mouse
*I*_D_	*t*_m_ (SD)	*t*_m_ (SD)	*I*_D_	*t*_m_ (SD)	*t*_m_ (SD)
1.80	10.88 (6.97)	0.74 (0.60)	2.11	8.56 (5.21)	0.73 (0.24)
2.10	10.69 (6.14)	0.55 (0.16)	2.21	8.16 (3.66)	0.66 (0.14)
2.30	10.48 (6.60)	0.58 (0.18)	2.30	9.60 (6.27)	0.69 (0.16)
3.20	13.79 (8.76)	0.72 (0.15)	3.21	12.78 (7.80)	0.86 (0.15)
4.10	18.25 (8.78)	0.86 (0.16)	4.10	18.73 (12.40)	1.06 (0.14)

**Table 2:** Mean throughput (bits/s) and standard deviation (in parentheses) across tested indexes of difficulty *I*_D_ (bits) for the BCI and the mouse at 1D and 2D tests.

1D	BCI	Mouse	2D	BCI	Mouse
*I*_D_	Throughput (SD)	Throughput (SD)	*I*_D_	Throughput (SD)	Throughput (SD)
1.80	0.32 (0.14)	4.91 (1.70)	2.11	0.47 (0.19)	4.78 (1.23)
2.10	0.35 (0.14)	6.31 (1.87)	2.21	0.45 (0.18)	5.39 (1.06)
2.30	0.39 (0.14)	6.37 (1.51)	2.30	0.45 (0.21)	5.30 (1.22)
3.20	0.38 (0.14)	6.28 (1.32)	3.21	0.42 (0.18)	5.23 (0.98)
4.10	0.33 (0.12)	6.26 (1.17)	4.10	0.37 (0.16)	5.12 (0.71)

**Table 3:** Contextual comparison of reported information-transfer values for the present study and selected related studies already cited in the manuscript. Direct quantitative equivalence is limited by differences in participant populations, task designs, device classes, and metric definitions.

Study	System(s)	Reported Value(s)	Task Characterization	Comparability Notes
Present study	Brainfingers BCI vs. Microsoft Optical Mouse	**Mean throughput (bits/s):** 1D: BCI 0.35, mouse 6.03; 2D: BCI 0.43, mouse 5.17	ISO/TS 9241-411-based 1D and 2D pointing tasks	Large non-disabled sample (n = 48); direct within-study comparison
Pino et al., 2003 [13]	Brain Actuated Technologies Cyberlink system vs. Logitech Cordless Wheel Mouse	**Throughput (bits/s):** BCI non-disabled users 0.182, BCI motion-impaired users 0.081; mouse non-disabled users 5.81, mouse motion-impaired users 1.12	One-directional and multidirectional ISO 9241-9-based tasks	Small mixed non-disabled/motion-impaired sample; older protocol; not directly equivalent to the present design
Nappenfeld and Giefing, 2018 [19]	g.tec medical engineering GmbH EEG-based BCI vs. Fujitsu Siemens optical computer mouse (and Saitek P990 joystick)	**Mean throughput (bits/s):** BCI 0.18; mouse 2.16; joystick 1.32	2D point-and-click cursor-control task	Five non-disabled participants; BCI click executed by the investigator after cursor acquisition
Molina-Cantero et al., 2021 [20]	NeuroSky MindWave single-channel EEG headset	**Average ITR:** 7 bits/min (≈0.117 bits/s)	2D pointer-control/target-selection task	ITR was estimated from a hypothetical 3 × 3 communication board, so it is not directly equivalent to ISO-style throughput
Kim et al., 2015 [21]	Emotiv Epoc EEG headset + custom-built eye tracker vs. mouse	**Overall ITR (bits/s):** hybrid 2.02–2.27; mouse 7.61	Multidirectional pointing-and-selection task	Eye tracking was used for pointing and BCI for selection; not reported in the same 1D/2D structure as the present study.
Hou et al., 2022 [22]	NextMind dry-electrode visually evoked EEG/SSVEP sensor	**Mean throughput (bits/s):** 0.82 (range: 0.58–1.17)	Fitts’ law target-activation/selection task	Six participants; no direct mouse comparator in the same experiment

## Data Availability

The data presented in this study are available from the corresponding author upon reasonable request. The data are not publicly available due to privacy and ethical restrictions.

## Abbreviations

The following abbreviations are used in this manuscript:

1D	One-dimensional
2D	Two-dimensional
AAC	Augmentative and alternative communication
ANOVA	Analysis of variance
BCI	Brain–computer interface
EEG	Electroencephalography
EMG	Electromyography
EOG	Electrooculography
GUI	Graphical user interface
HCI	Human–computer interaction
*I*_D_	Index of difficulty
*I*_De_	Effective index of difficulty
ITR	Information transfer rate
SD	Standard deviation
sEMG	Surface electromyography
SMR	Sensorimotor rhythm
